# High resolution metabolomics to discriminate compounds in serum of male lung cancer patients in South Korea

**DOI:** 10.1186/s12931-016-0419-3

**Published:** 2016-08-09

**Authors:** Aryo D. Pamungkas, Changyoung Park, Sungyong Lee, Sun Ha Jee, Youngja H. Park

**Affiliations:** 1 Metabolomics Laboratory, College of Pharmacy, Korea University, Sejong, 30019 Korea; 2 Korea University Guro Hospital 148, Gurodong-ro, Guro-gu, Seoul 08308 Korea; 3 Department of Epidemiology and Health Promotion and Institute for Health Promotion, Graduate School of Public Health, Yonsei University, Seoul, 03722 Korea

**Keywords:** High resolution metabolomics, Lung cancer, LC-MS, Biomarker, Bisphenol A, Retinol, L-proline

## Abstract

**Background:**

The cancer death rate escalated during 20th century. In South Korea, lung cancer is expected to contribute 12,736 deaths in men, the highest amount among all cancers. Several risk factors may increase the chance to acquiring lung cancer, with mostly related to exogenous compounds found in cigarette smoke and synthetic manufacturing materials. As the mortality rate of lung cancer increases, deeper understanding is necessary to explore risk factors that may lead to this malignancy. In this regard, this study aims to apply high resolution metabolomics (HRM) using LC-MS to detect significant compounds that might contribute in inducing lung cancer and find the correlation of these compounds to the subjects’ smoking habit.

**Methods:**

The comparison was made between healthy control and lung cancer groups for metabolic differences. Further analyses to determine if these differences are related to tobacco-induced lung cancer (past-smoker control vs. past-smoker lung cancer patients (LCPs) and non-smoker control vs. current-smoker LCPs) were selected. The univariate analysis was performed, including a false discovery rate (FDR) of *q* = 0.05, to determine the significant metabolites between the analyses. Hierarchical clustering analysis (HCA) was done to discriminate metabolites between the control and case subjects. Selected compounds based on significant *m/*z features of human serum then experienced MS/MS examination, showing that for many *m/z*, the patterns of ion dissociation matched with standards. Then, the significant metabolites were identified using Metlin database and features were mapped on the human metabolic pathway mapping tool of the Kyoto Encyclopedia of Genes and Genomes (KEGG).

**Results:**

Using metabolomics-wide association studies, metabolic changes were observed among control group and lung cancer patients. Bisphenol A (211.11, [M + H-H_2_O]^+^), retinol (287.23, [M + H]^+^) and L-proline (116.07, [M + H]^+^) were among the significant compounds found to have contributed in the discrimination between these groups, suggesting that these compounds might be related in the development of lung cancer. Retinol has been seen to have a correlation with smoking while both bisphenol A and L-proline were found to be unrelated.

**Conclusions:**

Two potential biomarkers, retinol and L-proline, were identified and these findings may create opportunities for the development of new lung cancer diagnostic tools.

**Electronic supplementary material:**

The online version of this article (doi:10.1186/s12931-016-0419-3) contains supplementary material, which is available to authorized users.

## Background

Cancer is a major public health problem that is currently one of the most leading causes of death in the United States. The cancer death rate escalated during the 20th century which was largely driven by rapid increases in lung cancer deaths as a consequence of tobacco epidemic. In 2015, the estimated number of cancer deaths in USA is dominated by lung cancer (27 % of all cancer deaths), surpassing prostate cancer for males (9 %) and breast cancer for females (15 %) [1]. In South Korea, meanwhile, lung cancer is expected to contribute 12,736 deaths in men, the highest amount among all cancers [2]. This increasing mortality rate in lung cancer patients has led physicians and scientists to seek a deeper understanding on this malignancy.

Lung cancer is a type of cancer that initially forms in lungs and can spread to nearby tissue even other organs, with the most recurrent metastatic sites were nervous system, bone, liver, respiratory system and adrenal gland [3]. Several risk factors may increase the chance to acquiring lung cancer, with mostly related to exogenous compounds found in cigarette smoke and synthetic manufacturing materials [4]. In fact, nine out of ten lung cancer cases are caused by smoking cigarettes [5]. Moreover, smokers have a greater risk for lung cancer today than they did in 1964, even though they smoke fewer cigarettes.

There are several diagnostic tools that are available in detecting lung cancer such as computed tomography (CT), magnetic resonance imaging (MRI), and positron emission tomography (PET) [6–8]. However, lung cancer patients do not experience signs and symptoms at the early stages of the disease which lead to late diagnosis—often when the cancer has already progressed. Furthermore, there are no initial screening methods available to diagnose lung cancer at its early stages.

A study mentioned that early detection of lung cancer can be performed by NMR-based metabolomics method for urine samples [9]. But the changes in metabolite levels in urine still cannot be correlated to compounds involved in lung cancer metabolism. Furthermore, metabolite levels in urine samples may vary depending on fluid intake prior to collection, and may also be affected by patients’ kidney function [10]. Miyamoto et al. (2015) conducted plasma samples analysis using GC-MS in determining metabolic changes of endogenous compounds in lung cancer patients [11]. However, the detection of exogenous compounds were not considered. Some other researches have dealt with respiratory diseases using NMR metabolomics like the study on electronic nose and exhaled breath [12], and on cystic fibrosis [13].

During the past decade, liquid chromatography coupled with mass spectrometry (LC-MS) has gone through huge development. The ability to determine thermolabile compounds directly without needing to undergo derivatization steps has given its advantage over gas chromatography (GC). With recently-developed configuration like quadrupole time-of-flight (Q-TOF), the range of screening has quickly expanded, enhancing both high mass resolution and mass fragmentation [14, 15].

Due to the disadvantages of urine samples mentioned earlier, this study aims to explore and identify significant compounds, exogenous and endogenous, that may induce cancer using LC-MS based high resolution metabolomics (HRM) on human serum samples from South Korean males for the purpose of providing early detection and non-invasive diagnosis of lung cancer. The significant compounds found to contribute in the discrimination between healthy and lung cancer subjects will also be confirmed and their correlation with smoking habit will be examined.

## Methods

### Samples collection

Serum samples were collected from Korean Cancer Prevention Study II, conducted from January 2004 to December 2004 across 11 hospitals in Seoul and Gyeonggi area, in permission of Ministry of Health and Welfare Korea. The total number of subjects participated in this study were 35,522 people which 105 subjects were selected for analysis. Of the 105 samples, 70 samples were from healthy people and 35 samples were from lung cancer patients which were diagnosed based on confirmed histologic diagnosis; details such as age and body mass index (BMI) are provided in Table 1. Basic parameters of the sample groups (age and BMI) were checked and was found to be statistically insignificant (*p* > 0.05). The study was approved by the Institutional Review Board of Yonsei University (2001-0029-011) and an informed consent was obtained from all participating patients.

**Table 1:** Age, Sex, Height, and Weight of Patients

Parameters	Control	Lung Cancer patients
Number of subjects	70	35
Age	60.60 ± 10.03	60.60 ± 10.10
Sex	Male 70	Male 35
Height (cm)	168.58 ± 5.51	165.94 ± 6.19
Weight (kg)	68.82 ± 8.05	64.77 ± 9.84
BMI (kg/m^2^)	24.20 ± 2.46	23.41 ± 2.57

Values are expressed as mean ± SD

### Sample preparation and LC-MS measurements

Fifty μl aliquots of samples were treated with acetonitrile (1:4, v/v), and centrifuged at 14,000 x g for 5 min at 4 °C for protein separating [16]. Samples were analyzed using Ultra Performance Liquid Chromatography system (Agilent 1260 Infinity Quaternary, Agilent, Santa Clara, CA, USA) with iFunnel Q-TOF mass spectrometry (Agilent Q-TOF 6550, Agilent, Santa Clara, CA, USA) in duplicates. This LC-MS/MS is ideally suited for metabolic stability and profiling studies, since this system has the highest sensitivity to detect compounds at low pg/mL levels and 40 k resolving power to determine excellent mass and isotope accuracy for confident identification of metabolites.

An electrospray interface was operated in positive ion mode. The conditions for the acquisition parameters were: gas temperature 250 °C, drying gas 14 ml/min, nebulizer pressure 35 psig, sheath gas temperature 250 °C and sheath gas flow 11 ml/min [17]. Mobile phase A consisted of 0.1 % formic acid in water (HPLC grade, Tedia, Ohio, USA) and mobile phase B was 0.1 % formic acid in acetonitrile (HPLC grade, Tedia, Ohio, USA). The gradient was programed as follows: 0–1 min: 5 % for B, 1–9 min: gradient increase to 5 % for B, 9–12 min: hold 56 % for B, 12–13.5 min: 90 % for B; 13.5–13.6 min: hold 5 % for B. The column heater was adjusted at 30 °C. The flow rate was 0.4 L/min. Three microliter of sample was injected on to a 1.9 μm, 100x2.1 mm C18 column (Thermo, Waltham, USA) [18]. Detection of *m/z* of ions from 50 to 1000 with 20,000 resolution over 15 min LC runs with data extraction using apLCMS [19] provides a minimum of 3000 reproducible features, many with sufficient mass accuracy to allow prediction of elemental composition. Ion intensity, *m/z*, and retention time defined an *m/z* feature. Meanwhile, selected compounds based on significant *m/*z features of human serum then experienced MS/MS examination using LC-MS triple quad (QQQ) 6490 (Agilent, CA, USA), showing that for many *m/z*, the patterns of ion dissociation matched with standards.

### Metabolic profiling with univariate and multivariate statistical analysis

After running high resolution, accurate mass system of Q-TOF, raw data were processed using apLCMS producing the total features from the samples for the subsequent analyses. In performing statistical analyses and bioinformatics, features from duplicate LC-MS analyses were averaged, log_2_ transformed and normalized using z-transformation. It included univariate analysis, Manhattan plot, and false discovery rate (FDR) [20] using Limma R package [21] to determine the significant metabolites between healthy controls and lung cancer patients. Additionally, the metabolic profiles were discriminated using two-way hierarchical cluster analysis (HCA) to separate two groups in connection with metabolites [22]. Limma is originally a package for the analysis of gene expression data arising from microarray or RNA-Seq technologies. It provides the ability to compare between many targets simultaneously [23, 24]. Moreover, classification of samples based on significant metabolites levels was done using receiver operating characteristic (ROC) curves (MedCalc).

### Comparison groups

**Fig. 1:**
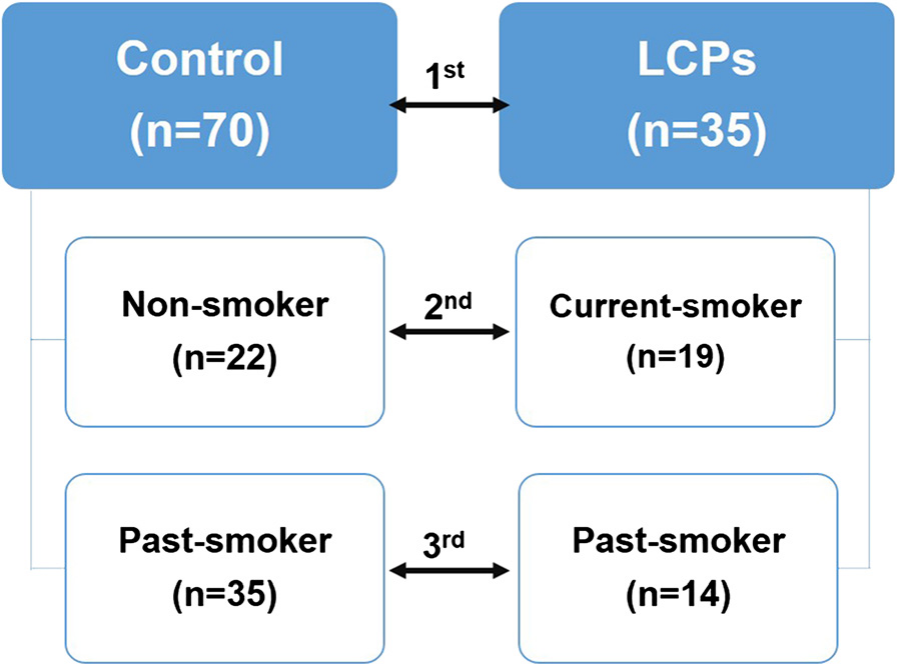
Scheme of study design. Comparison groups used in discriminating significant compounds that may be linked in lung cancer induction

A total of three analyses were conducted in this study, as summarized in Fig. 1. Initially, the comparison was made between healthy control (*n* = 70) and lung cancer patients (LCPs) (*n* = 35) for metabolic differences (shown as 1st analysis). Then, further analyses were performed based on smoking history of the subjects: non-smoker control vs. current-smoker LCPs (2nd analysis) and past-smoker control vs. past-smoker LCPs (3rd analysis). The 2nd analysis was conducted to emphasize whether the findings have correlation with smoking habit while the 3rd analysis determined whether the findings demonstrated no association with smoking habit.

### Data annotation and pathway analysis

The significant metabolites were then annotated using Metlin Metabolite Database [25]. These metabolites were listed in a batch containing their *m/z* value, name and formula, which could be sorted based on the availability of Kyoto Encyclopedia of Genes and Genomes (KEGG) number. Using this number, the metabolites were queried into the human metabolic pathway [26]. Detected features that matched known human intermediary metabolites are shown as black dots in the map.

## Results

### Manhattan plot and two-way hierarchical cluster analysis (HCA)

Using metabolomics-wide association studies (MWAS), metabolic changes in lung cancer patients were determined. A Manhattan plot utilizes statistical tests to visually identify significant features which are differentially expressed in subjects when compared to a control group. FDR adjustment, a multiple testing correction method which adjusts *p*-values (*q*-values) to reduce the occurrences of false positives was also used for optimum statistical significance.

**Fig. 2:**
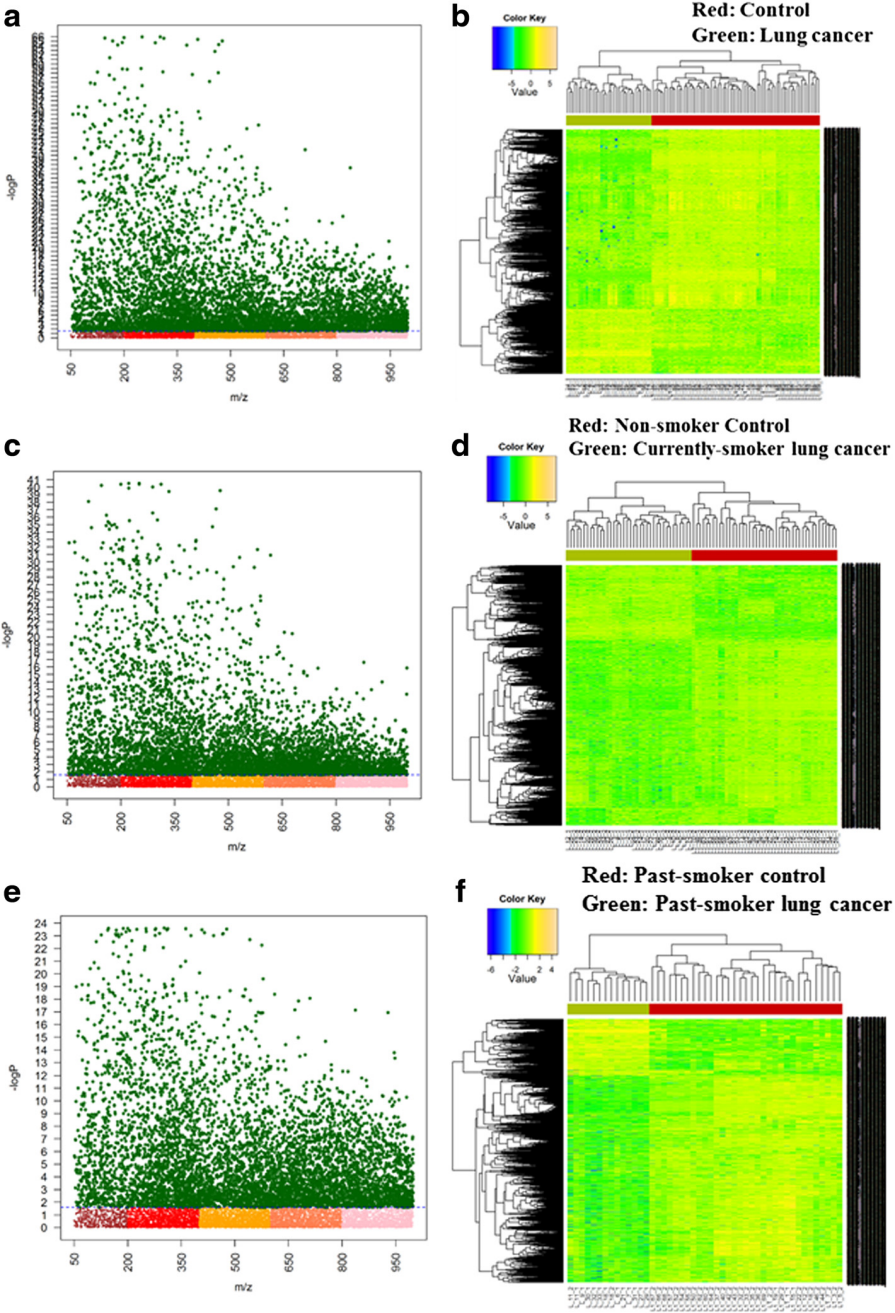
Metabolome-wide association study (MWAS). Manhattan plot (**a**, **c**, **e**) and HCA (**b**, **d**, **f**) using FDR significant features of: (**a**–**b**) control vs LCPs, (**c**–**d**) non-smoker control vs current-smoker LCPs and (**e**–**f**) past-smoker control vs past-smoker LCPs

The Y axis represents the –log10 of the raw *p*-value between two compared groups while the X axis are *m/z* values ranging from 50 to 1000 (Fig. 2). The dashed line shows the FDR significant threshold (*q* = 0.05) which separates the significant features from other insignificant *m/z*. In this regard, all metabolites which lie above this threshold are considered statistically significant from the control group.

In control vs LCPs analysis, the significant features were 6096 out of the 9516 total detected features, as shown in Fig. 2a. On the other hand, 5727 significant features were seen from the 11,234 total features in non-smoker control vs current-smoker LCPs group (Fig. 2c) while 6289 significant features out of the 12,349 total features were detected in past-smoker control vs past-smoker LCPs analysis (Fig. 2e). Furthermore, two-way HCA was performed to identify which metabolites are the most important for sample grouping. A clear separation of the control and case groups is expected in a dataset with distinct components. As seen in Fig. 2 (b, d, and f), the control group which is represented by the red panel is grouped as one cluster while the others (green panel) are on the other side of the clustering. This apparent separation in the heatmaps suggest that the metabolites are highly differentiated from the control.

### KEGG pathway analysis

**Fig. 3:**
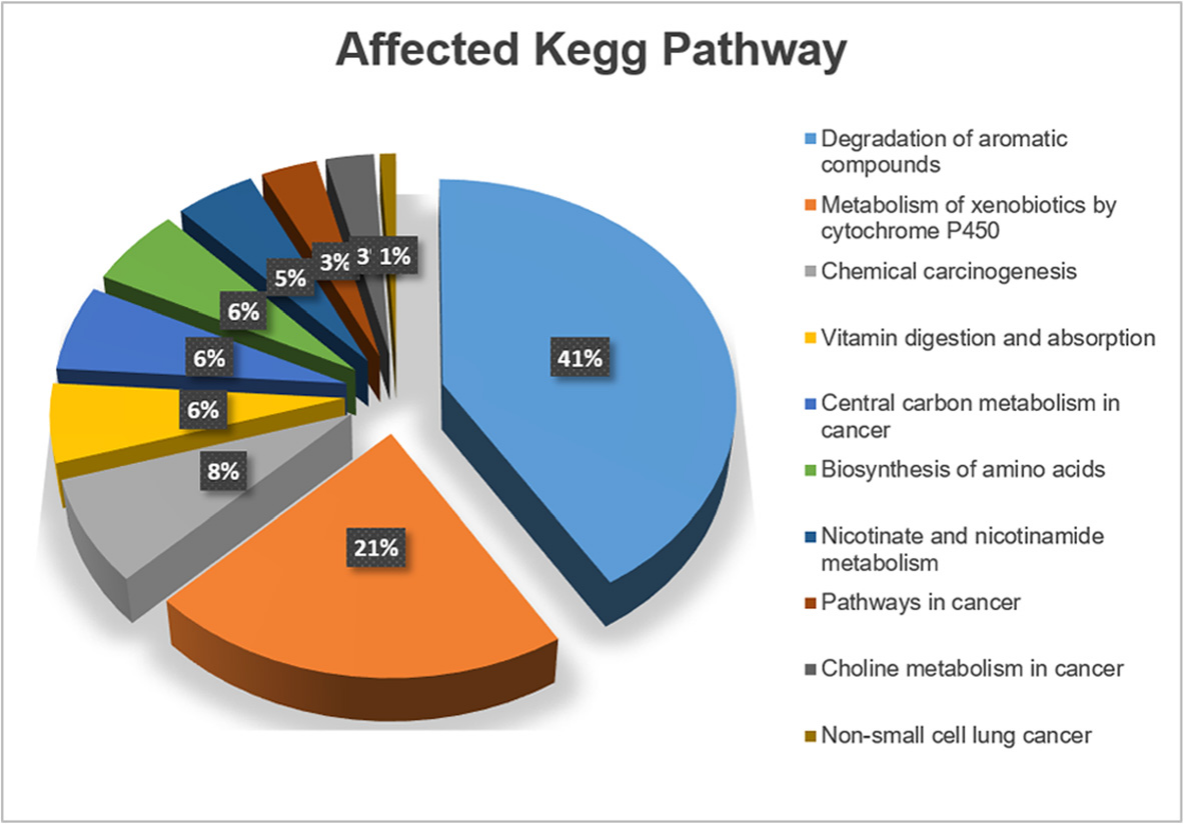
KEGG mapping. Possible affected pathways from the compounds discriminating control vs LCPs

**Fig. 4:**
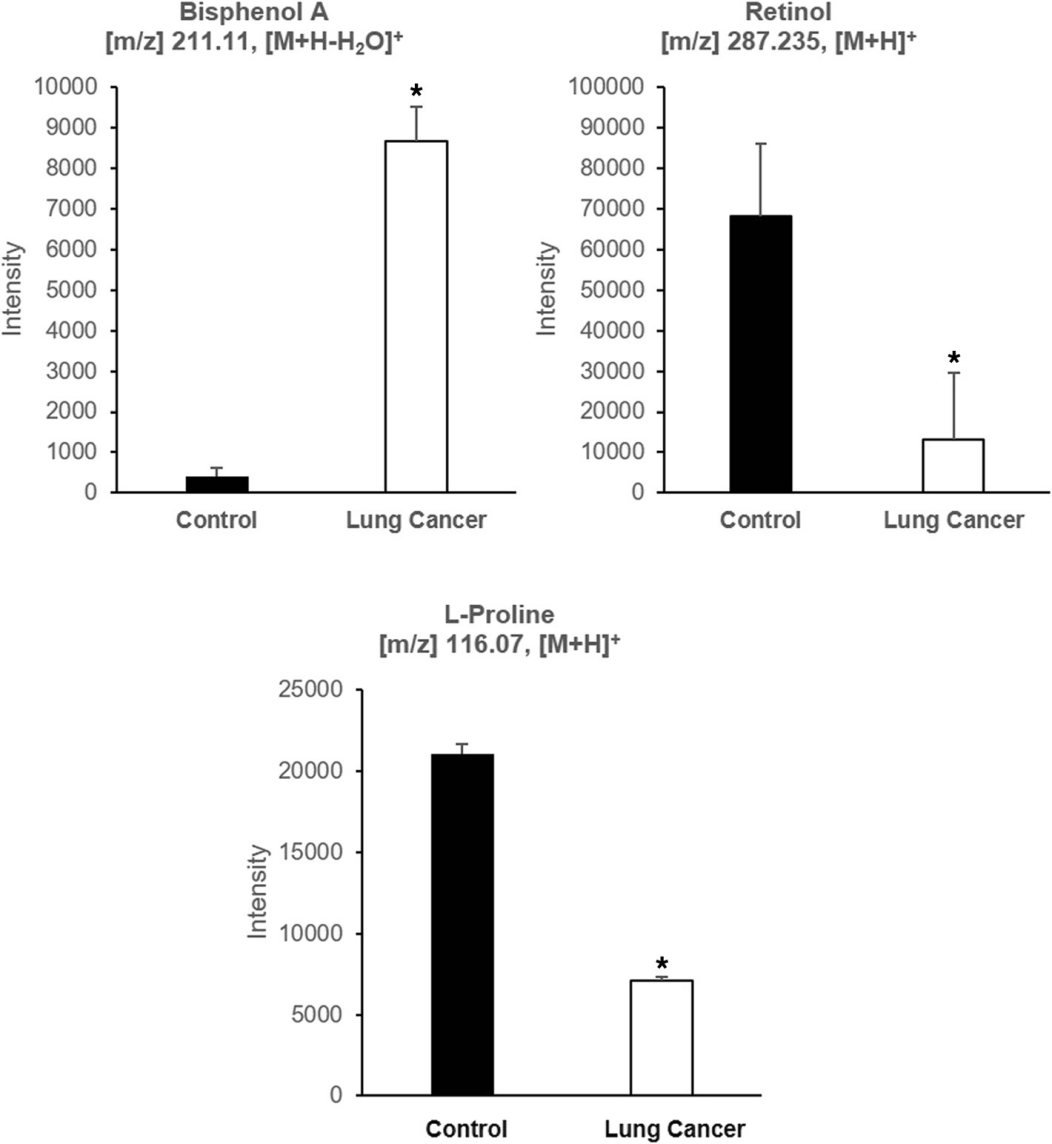
The abundance of selected compounds in control vs LCPs analysis. Relative concentrations of Bisphenol A (**a**), Retinol (**b**), and L-proline (**c**). *shows significant difference (*p* < 0.05)

Significant features were annotated in Metlin database using positive ion adducts (M + H, M + Na, and M+ H_2_O). After data processing to include only the ones with KEGG numbers, the human metabolic pathway was used to determine the possible pathways that were affected. In the KEGG pathway map, the black dots represent the compounds found in the human metabolic pathway that were statistically different between control and LCPs, as shown in Additional file 1. These compounds are part of different metabolic pathways in humans that are potentially affected by lung cancer. Figure 3 shows the top ten pathways showing the percentage of the number of metabolites versus the total number of compounds in these pathways. Upon entering the body, xenobiotic compounds which are usually lipid soluble, are generally not metabolized easily by digestive enzymes and therefore, may not be excreted fully. The liver then performs biotransformation to eliminate these compounds, thus, it can be predicted that the degradation of aromatic compounds and metabolism of xenobiotic compounds are two of the top affected pathways which can be related to these exogenous compounds that were detected in the samples. In addition, chemical carcinogenesis pathway is one of the notable results since large amount of xenobiotics is reported as carcinogen [4]. Pathway analysis also showed that vitamin digestion and biosynthesis of amino acids were also disrupted in patients with lung cancer. Due to their capability to cause metabolic disturbances (as shown in KEGG pathway), bisphenol A (BPA), retinol, and L-proline were selected to be the best candidates to undergo verification and further analysis. The relative concentrations of these compounds are shown in Fig. 4 ROC analysis was applied to assess how well the levels of these compounds could classify LCPs from controls. For BPA, the area under the curve (AUC) of 0.93 was acquired, and for retinol and L-proline, the corresponding result was 0.72 and 0.95 (as shown in Additional file 2).

### Identification and verification of potential biomarkers

Xenobiotic and endogenous compounds were identified between control and lung cancer subjects, supported by multivariate statistical analysis. BPA was found to be elevated in cancer subjects, whereas retinol and L-proline were found to be lowered. These compounds were considered significant in the groups shown in Table 2. Validation of these compounds was performed by multiple reaction monitoring in positive mode to detect the specific precursor to product ion transitions: *m/z* 226.9 → *m/z* 185.9 for BPA, *m/z* 286.2 → *m/z* 245 for retinol and *m/z* 116 → *m/z* 69.9 for L-proline. The MS/MS data can be seen in Additional files 3, 4, 5, 6, 7, and 8.

**Table 2:** Main compounds contributing for the discrimination in each comparison group

Compounds	Comparison groups		
	Control (*n* = 70)	Non-smoker (*n* = 22)	Past-smoker (*n* = 35)
	LCPs (*n* = 35)	Current-smoker (*n* = 19)	Past-smoker (*n* = 14)
BPA	✓		✓
Retinol	✓	✓	
L-proline	✓	✓	✓

## Discussion

This study explored lung cancer-specific low molecular weight compounds that may have potential roles in inducing the disease and can be used as candidate biomarkers for the early diagnosis of lung cancer. Using serum samples from all patients, three compounds: BPA, retinol, and L-proline were identified as statistically significant compounds and were correlated to the subjects’ smoking habit.

BPA is used in the production of polycarbonate plastics which is the main ingredient of many manufactured goods such as toys, drinking containers, food cans, eyeglass lenses and electronic appliances [27–29]. It was found to be significant only in past smoker control vs. past smoker LCPs analysis (as shown in Additional file 9). With this finding, it is possible that this compound may induce lung cancer but it is not related to the smoking history of the subjects. Since it was detected insignificant under non-smoker control vs. current-smoker LCPs analysis group, it can be inferred that BPA may have come from other sources (e.g. plastic products, food cans) and not from cigarettes. Previous studies have mentioned several possible roles of BPA in carcinogenesis [30–32]. Although having a BPA concentration of less than 10^−4^ M had not triggered the proliferation process of lung cancer cells, it had stimulated in vitro migration and invasion of the cells via up-regulation of matrix metalloproteinase-2 which could enhance the susceptibility to carcinogenesis. Another study showed that BPA could alter Peroxisome Proliferator-activated Receptors (PPARs), in this case, the PPARγ. This ligand-activated transcription factor was found to induce differentiation and apoptosis in lung cancer cells. As its antagonist, BPA promoted prevention of apoptosis, resulting the survival of cancer cells [30–32]. In addition, BPA concentration in human samples was correlated with intracranial tumor [33] and prostate cancer [34].

Vitamin A, on one hand, is also one of the selected metabolites observed to be differentially expressed in the comparison groups. This vitamin comes from carotenoid and retinoid. Basically, carotenoids are known for their antioxidant effects and are derived from plants [35]. Retinoid are physiological regulators of embryonic development, vision, reproduction, bone formation, hematopoiesis, differentiation, proliferation and apoptosis [36]. Retinol was found to be effective against breast, prostate, and ovarian cancers in animal treatment models. Also, the ability of retinol to induce apoptosis suggested that it is potential in prevention and treatment of lung cancer and other types of cancer [37]. In this study, detection of retinol was significantly less in current-smoker patients as compared to non-smoker control (as shown in Additional file 9). Therefore, it is suggested that the level of retinol in the body may have been decreased in patients who smoke, thus increasing their risks in having the disease. In addition, using control and LCPs comparison, KEGG mapping displayed vitamin digestion and absorption as one of the affected human pathway. This finding was consistent with previous reports. Cigarette smoke could induce depletion of retinol serum levels in rats, as demonstrated by Li et al. [38]. It was further investigated to be correlated with the development of emphysema. In addition, Yuan et al. revealed that low level of serum retinol is associated with the increase of lung cancer risk in China population [39].

Meanwhile, L-proline, a non-essential amino acid, was found to be lowered in LCPs, confirming a previously reported study [40]. Zhao et al. reported that L-proline was one of the amino acid which its plasma concentration was found to be decreased in LCPs, compared with control group (*p* < 0.001). Rapid increase in the transcription of proline dehydrogenase by tumor suppressor p53 triggered the degradation of this amino acid in cancer [41]. In addition, using control and LCPs comparison, KEGG mapping displayed biosynthesis of amino acids as one of the affected human pathway. As shown in Additional file 9, the decreasing L-proline occurred in all parameters that were analyzed, suggesting that this compound is possible to act as biomarker for lung cancer, regardless of smoking habit.

## Conclusions

In summary, two potential biomarkers, retinol and L-proline, were identified using HRM with the combination of pathway analysis from significant metabolites that were found in serum samples of Korean male LCPs. It was also seen that one of them was correlated to smoking habit of LCPs. In addition, one exogenous compound, BPA, which has not been linked yet to lung cancer patients was demonstrated as significant feature that contributed to discriminate healthy and LCPs groups, regardless of smoking habit. These findings may create opportunities for the development of new lung cancer diagnostic tools. In the future, correlations to disease type, stage, also the applicability to female test subjects group may also be conducted. Also, further study using larger population should be conducted.

## Abbreviations

BPA, bisphenol A; FDR, false discovery rate; HCA, hierarchical cluster analysis; HRM, high resolution metabolomics; KEGG, kyoto encyclopedia of genes and genomes; LCPs, lung cancer patients; Q-TOF, quadrupole time-of-flight

## Additional files

Additional file 1: Kyoto Encyclopedia Genes and Genomics (KEGG) pathway in control vs LCPs. This figure shows the mapping of matched features covering human metabolites. (TIF 1915 kb) Additional file 2: Classification of the subjects using significant compounds levels. ROC curves showed how the levels of BPA, retinol and L-proline could classify LCPs from controls. (PDF 130 kb) Additional file 3: Qualitative analysis of BPA from its total ion chromatogram. A) from serum sample, B) from standard. (TIF 1214 kb) Additional file 4: The similarity of BPA fragmentation pattern. A) MS/MS on serum sample, B) MS/MS on itself. Collision energy of 0 and 5 were used. (TIF 1719 kb) Additional file 5: Qualitative analysis of retinol from its total ion chromatogram. A) from standard, B) from serum sample. (TIF 1046 kb) Additional file 6: Qualitative analysis of L-proline from its total ion chromatogram. A) from standard, B) from serum sample. (TIF 985 kb) Additional file 7: The similarity of retinol fragmentation pattern. A) MS/MS on itself, B) MS/MS on serum sample. Collision energy of 0, 10, 20 and 30 were used. (TIF 3088 kb) Additional file 8: The similarity of L-proline fragmentation pattern. Fragmentation can be compared from standard and serum sample with collision energy of 0 (A, B), 10 (C, D), and 20 (E, F). (TIF 2236 kb) Additional file 9: Relative concentrations of three compounds in 2nd and 3rd analysis. BPA, with its increasing abundance, was detected only in 3rd analysis while retinol, with its decreasing abundance, was detected only in 2nd analysis. L-proline was found to be lowered in both 2nd and 3rd analysis. *shows significant difference (*p* < 0.05) (TIF 1390 kb)
