# Supplementary figures and images for: High resolution metabolomics to discriminate compounds in serum of male lung cancer patients in South Korea
Source: Respir Res. 2016 Aug 9;17:100. doi: 10.1186/s12931-016-0419-3 (PMC4977704; doi:10.1186/s12931-016-0419-3)

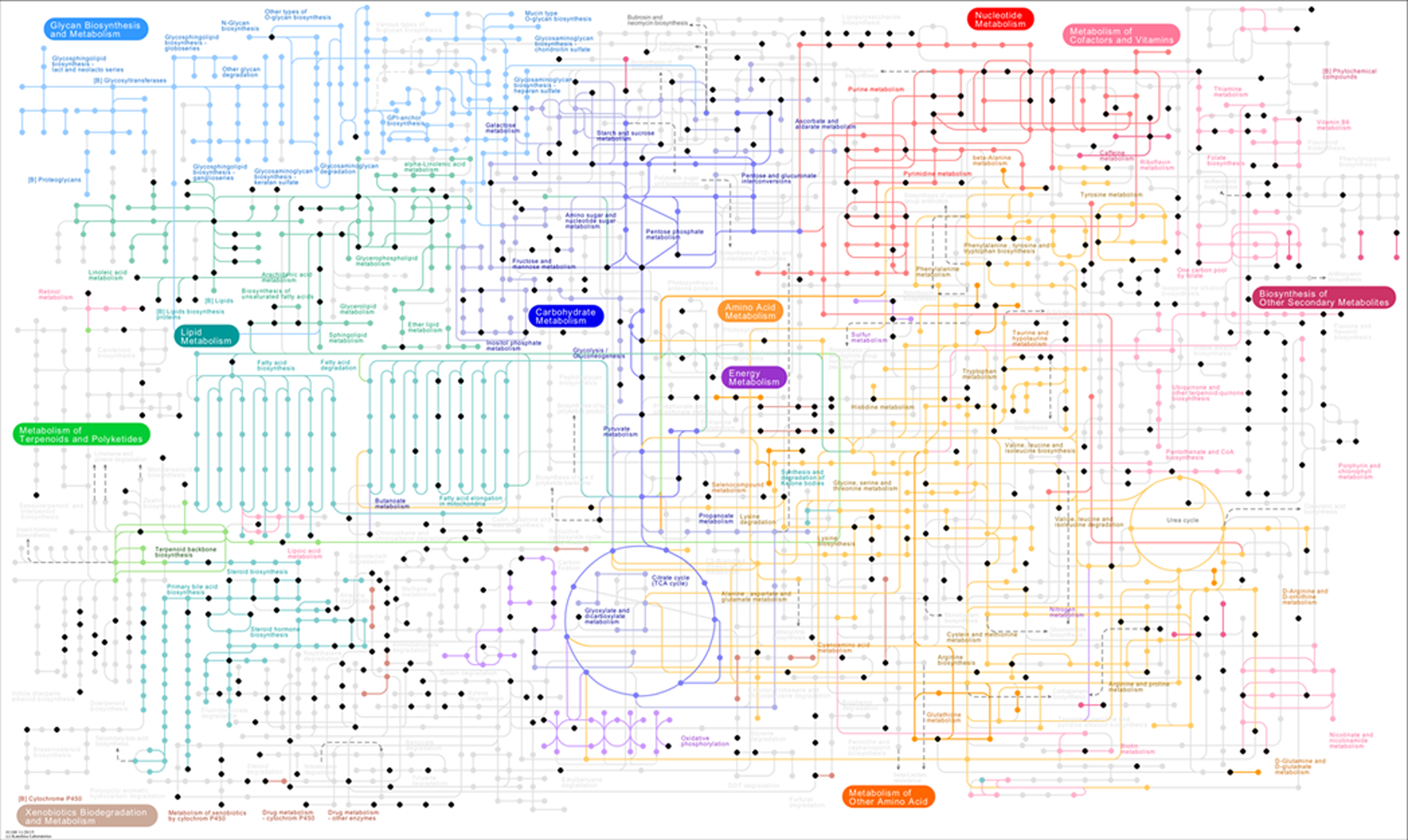

Supplement: Additional file 1: — Kyoto Encyclopedia Genes and Genomics (KEGG) pathway in control vs LCPs. This figure shows the mapping of matched features covering human metabolites. (TIF 1915 kb) [file 12931_2016_419_MOESM1_ESM.tif]

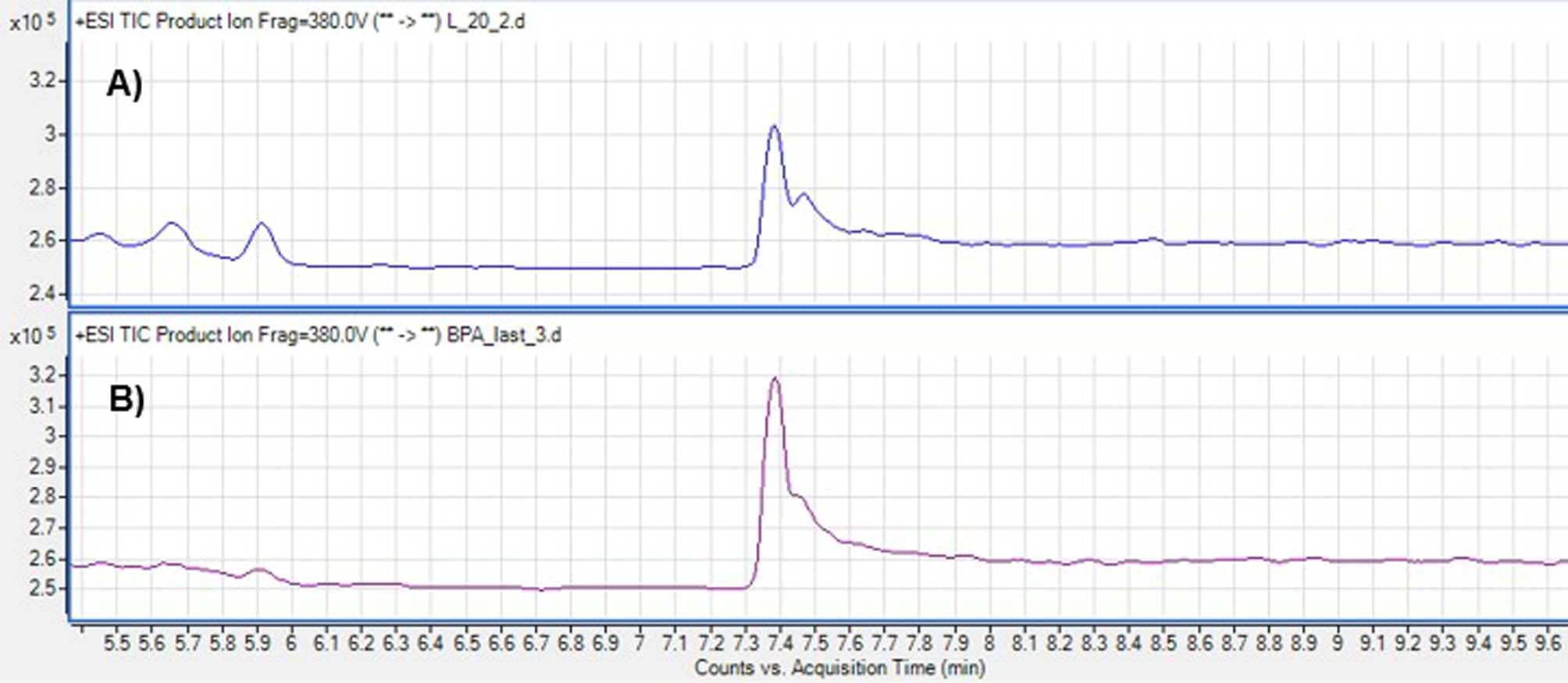

Supplement: Additional file 3: — Qualitative analysis of BPA from its total ion chromatogram. A) from serum sample, B) from standard. (TIF 1214 kb) [file 12931_2016_419_MOESM3_ESM.tif]

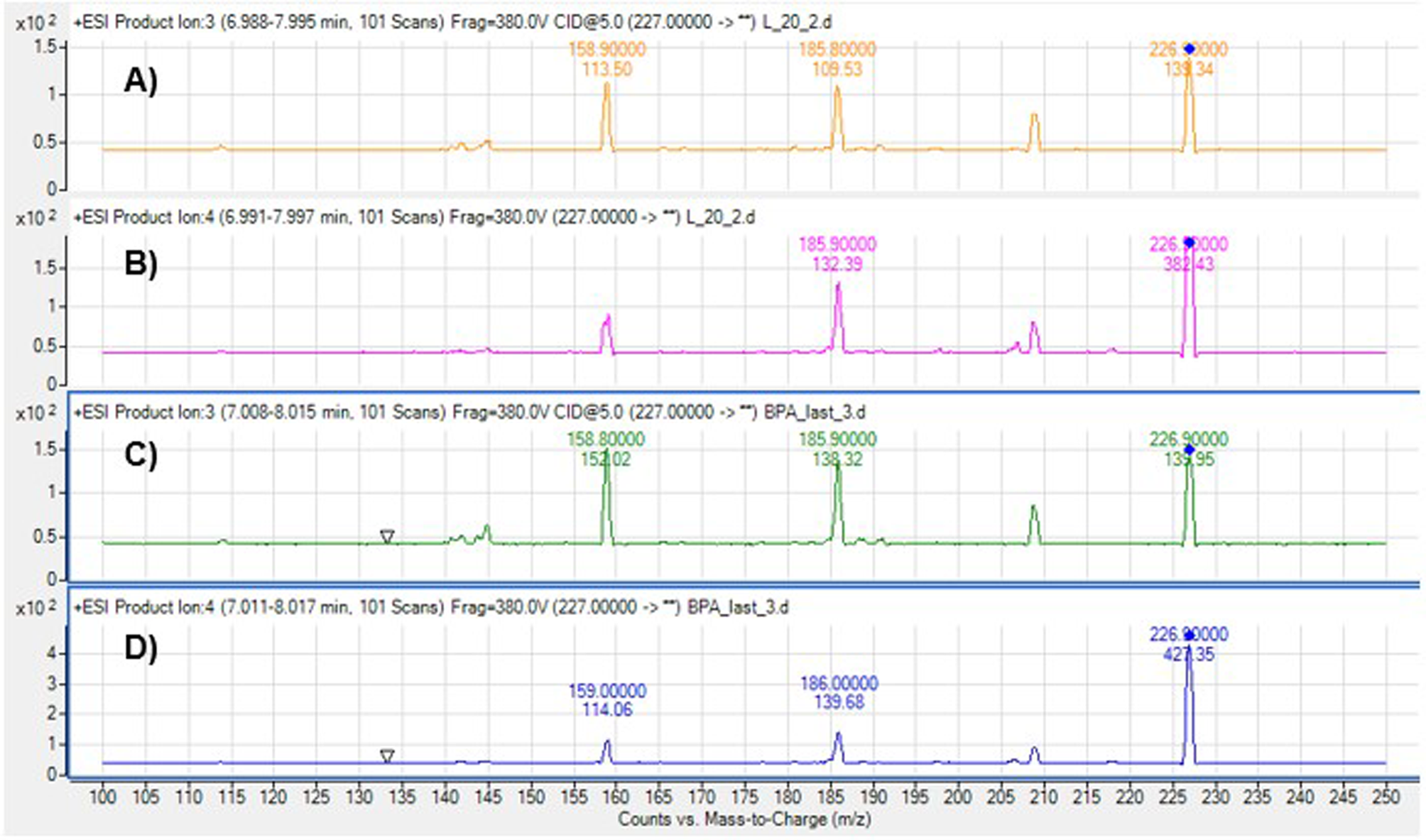

Supplement: Additional file 4: — The similarity of BPA fragmentation pattern. A) MS/MS on serum sample, B) MS/MS on itself. Collision energy of 0 and 5 were used. (TIF 1719 kb) [file 12931_2016_419_MOESM4_ESM.tif]

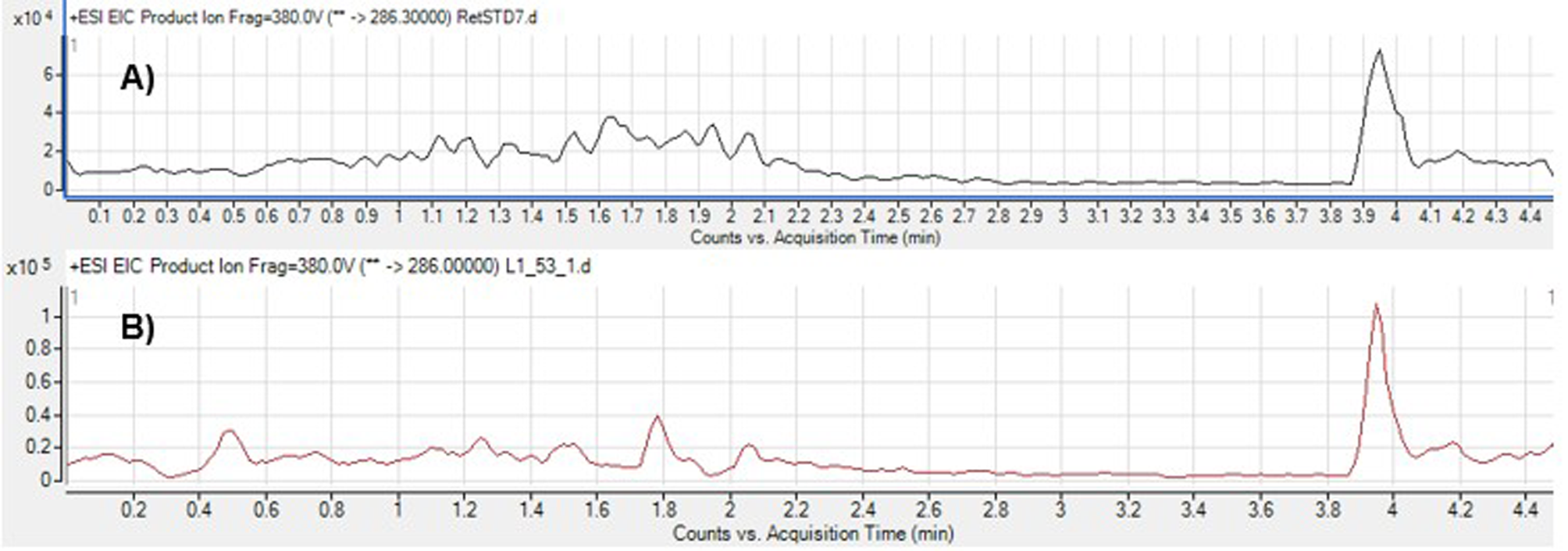

Supplement: Additional file 5: — Qualitative analysis of retinol from its total ion chromatogram. A) from standard, B) from serum sample. (TIF 1046 kb) [file 12931_2016_419_MOESM5_ESM.tif]

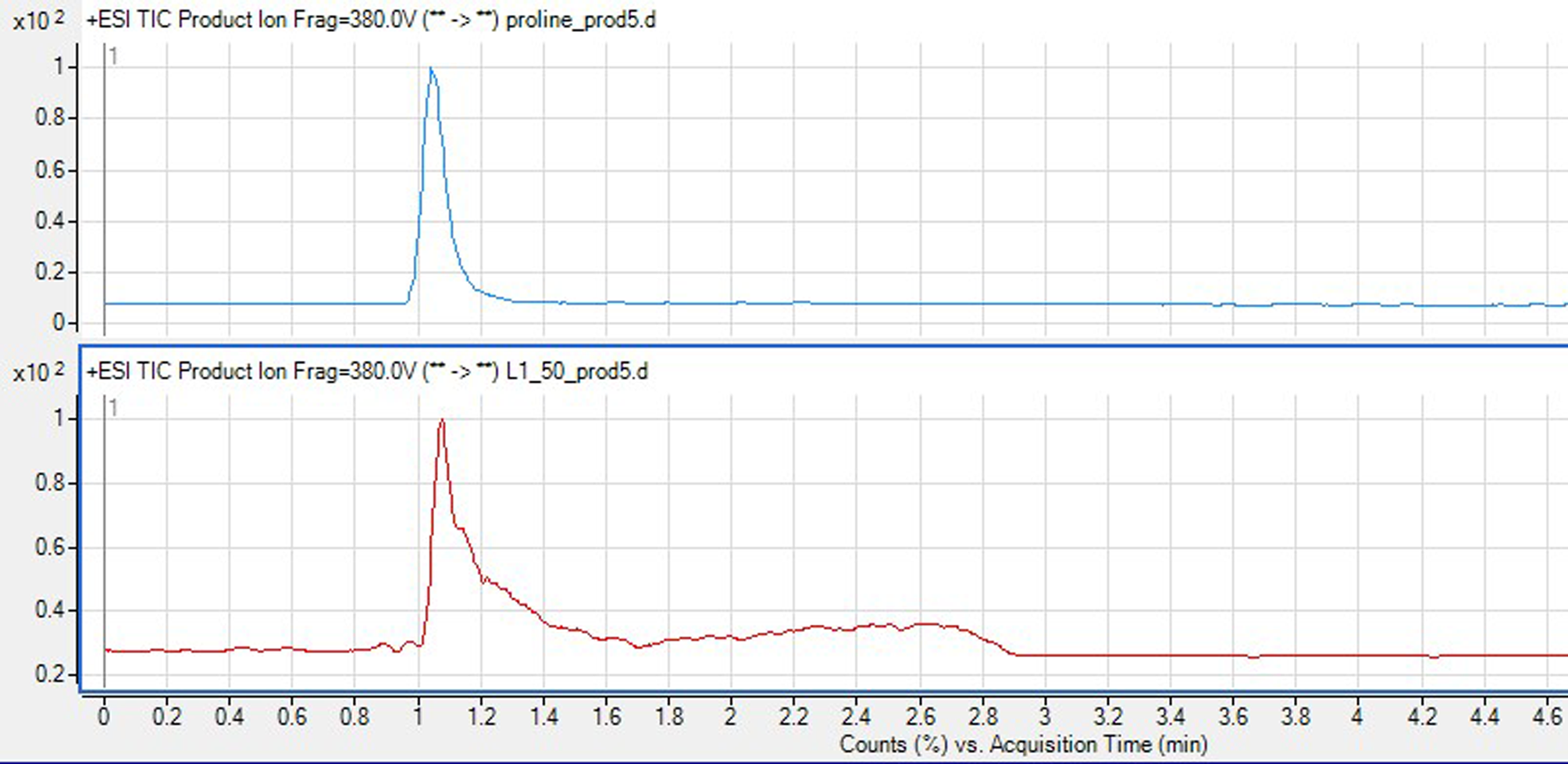

Supplement: Additional file 6: — Qualitative analysis of L-proline from its total ion chromatogram. A) from standard, B) from serum sample. (TIF 985 kb) [file 12931_2016_419_MOESM6_ESM.tif]

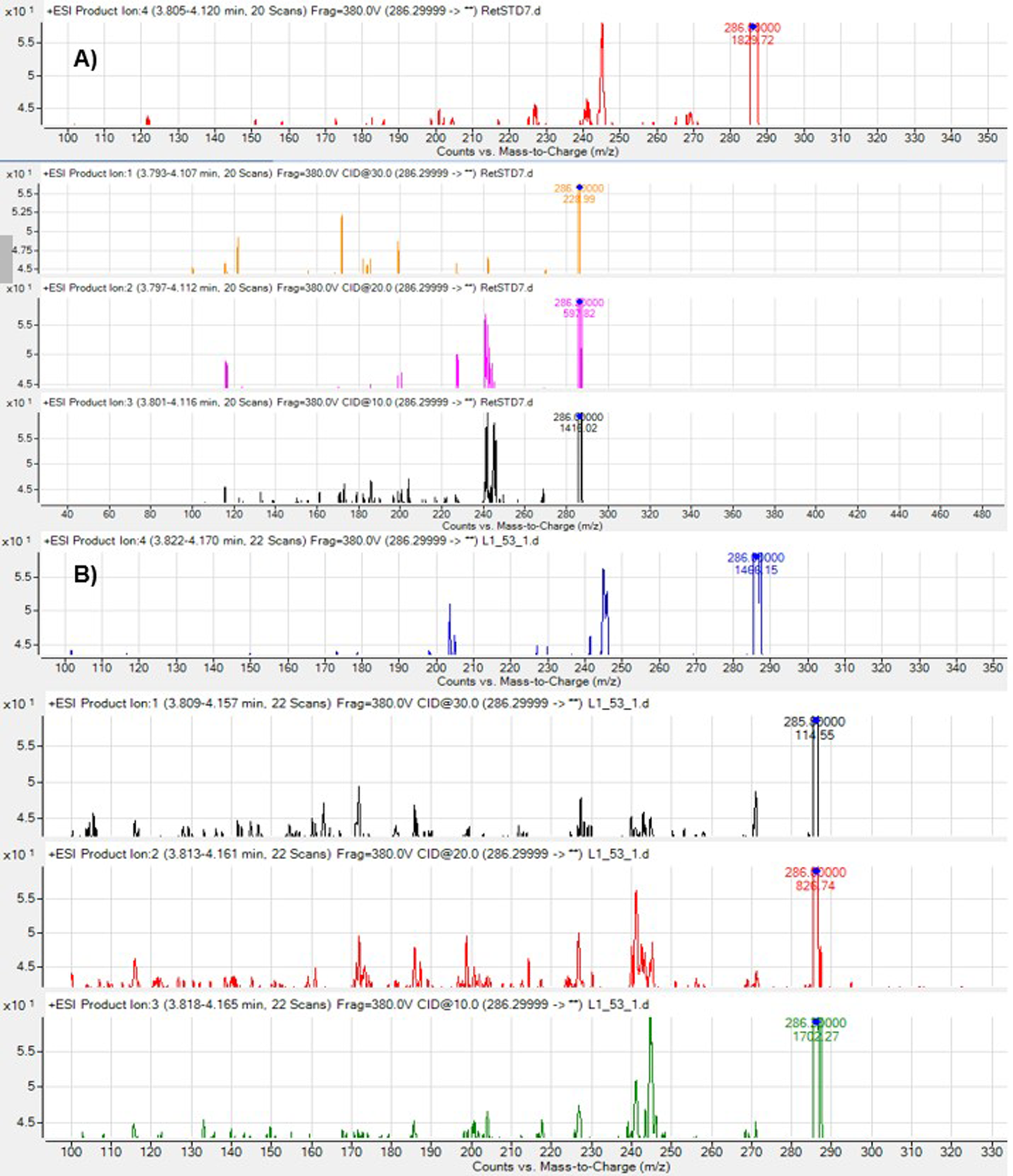

Supplement: Additional file 7: — The similarity of retinol fragmentation pattern. A) MS/MS on itself, B) MS/MS on serum sample. Collision energy of 0, 10, 20 and 30 were used. (TIF 3088 kb) [file 12931_2016_419_MOESM7_ESM.tif]

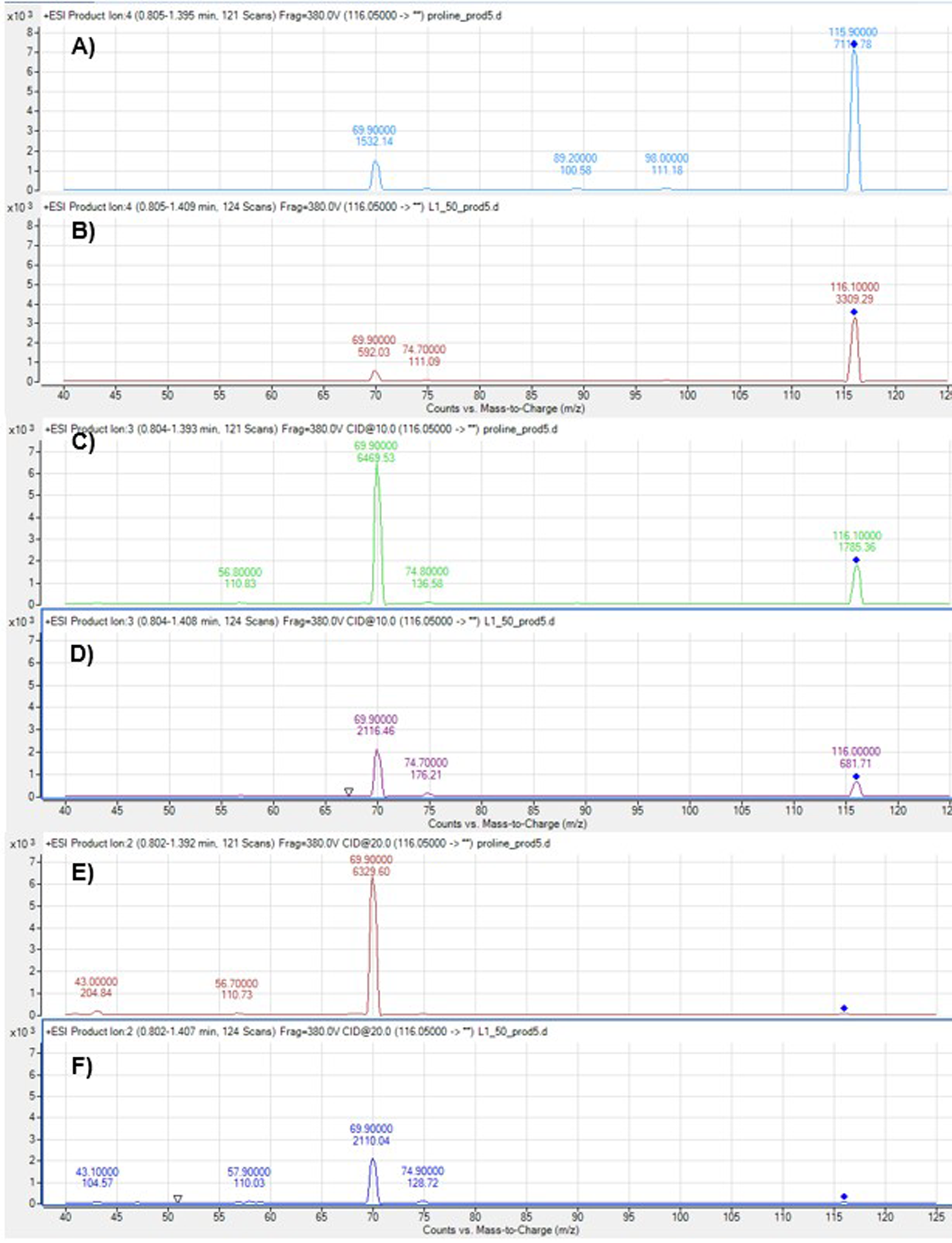

Supplement: Additional file 8: — The similarity of L-proline fragmentation pattern. Fragmentation can be compared from standard and serum sample with collision energy of 0 (A, B), 10 (C, D), and 20 (E, F). (TIF 2236 kb) [file 12931_2016_419_MOESM8_ESM.tif]

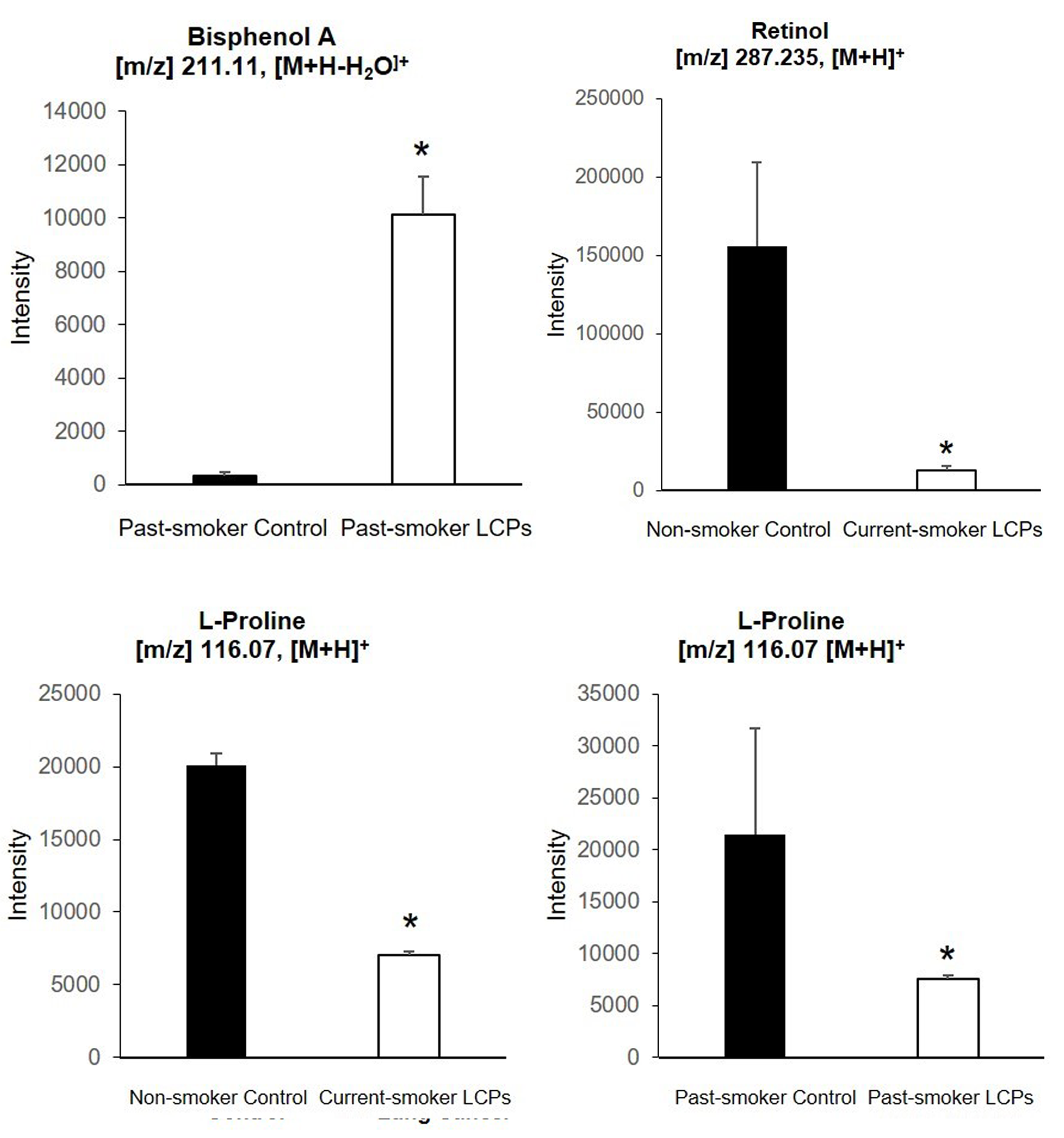

Supplement: Additional file 9: — Relative concentrations of three compounds in 2nd and 3rd analysis. BPA, with its increasing abundance, was detected only in 3rd analysis while retinol, with its decreasing abundance, was detected only in 2nd analysis. L-proline was found to be lowered in both 2nd and 3rd analysis. *shows significant difference (p < 0.05) (TIF 1390 kb) [file 12931_2016_419_MOESM9_ESM.tif]
