# Supplementary material for: High resolution metabolomics to discriminate compounds in serum of male lung cancer patients in South Korea
Source: Respir Res. 2016 Aug 9;17:100. doi: 10.1186/s12931-016-0419-3 (PMC4977704; doi:10.1186/s12931-016-0419-3)
Supplement: Additional file 2: — Classification of the subjects using significant compounds levels. ROC curves showed how the levels of BPA, retinol and L-proline could classify LCPs from controls. (PDF 130 kb) [file 12931_2016_419_MOESM2_ESM.pdf]

Additional file 2. Classification of the subjects using significant compounds levels.

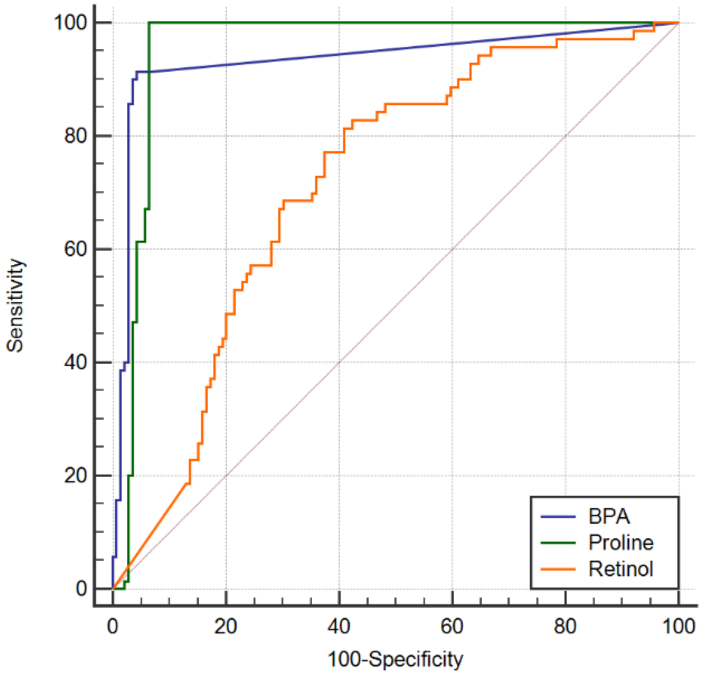

ROC curves showed how the levels of BPA, retinol and L-proline could classify LCPs from controls.

<u>BPA:</u>

Sensitivity: 91.4%	+Predictive Value: 91.4
Specificity: 95.7%	-Predictive Value: 95.7

<u>Retinol:</u>

Sensitivity: 81.4%	+Predictive Value: 50
Specificity: 59.0%	-Predictive Value: 86.3

<u>Proline:</u>

Sensitivity: 100%	+Predictive Value: 88.6
Specificity: 93.5%	-Predictive Value: 100.0
